# Anti-Growth, Anti-Angiogenic, and Pro-Apoptotic Effects by CX-4945, an Inhibitor of Casein Kinase 2, on HuCCT-1 Human Cholangiocarcinoma Cells via Control of Caspase-9/3, DR-4, STAT-3/STAT-5, Mcl-1, eIF-2α, and HIF-1α

**DOI:** 10.3390/ijms23116353

**Published:** 2022-06-06

**Authors:** Saini Wang, Anil Kumar Yadav, Jin-Yi Han, Keun Soo Ahn, Byeong-Churl Jang

**Affiliations:** 1 Department of Molecular Medicine, College of Medicine, Keimyung University, 1095 Dalgubeoldaero, Dalseo-gu, Daegu 42601, Korea; saini0920@naver.com (S.W.); yadav127@umn.edu (A.K.Y.); 2 Department of Surgery, Keimyung University Dongsan Hospital, 1035 Dalgubeol-daero, Dalseo-gu, Daegu 41931, Korea; esr0319@naver.com; 3 The Hormel Institute, University of Minnesota, Austin, MN 55812, USA

**Keywords:** CX-4945, CK2, STAT-3, Mcl-1, HIF-1α, HuCCT-1

## Abstract

Overexpression of casein kinase 2 (CK2) has an oncogenic and pro-survival role in many cancers. CX-4945 (Silmitasertib) is a CK2 inhibitor with anti-cancerous and anti-angiogenic effects. Up to date, the anti-cancer effect and mechanism of CX-4945 on human cholangiocarcinoma (CCA) remain unclear. This study investigated whether CX-4945 inhibits growth and induces apoptosis of HuCCT-1 cells, a human CCA cell line. Of note, treatment with CX-4945 at 20 μM markedly reduced survival and induced apoptosis of HuCCT-1 cells, as evidenced by nuclear DNA fragmentation, PARP cleavage, activation of caspase-9/3, and up-regulation of DR-4. Although CX-4945 did not affect the phosphorylation and expression of CK2, it vastly inhibited the phosphorylation of CK2 substrates, supporting the drug’s efficacy in inhibiting CK2 and its downstream pathway. Importantly, knockdown of CK2 that partially suppressed the phosphorylation of CK2 substrates resulted in a significant reduction of HuCCT-1 cell survival. In addition, CX-4945 reduced the phosphorylation and expression of STAT-3 and STAT-5 in HuCCT-1 cells, and pharmacological inhibition or respective knockdown of these proteins resulted in significant growth suppression of HuCCT-1 cells. CX-4945 also had abilities to decrease Mcl-1 expression while increasing eIF-2α phosphorylation in HuCCT-1 cells. Furthermore, there was a time-differential negative regulation of HIF-1α expression by CX-4945 in HuCCT-1 cells, and knockdown of HIF-1α caused a significant reduction of the cell survival. In summary, these results demonstrated that CX-4945 has anti-growth, anti-angiogenic, and pro-apoptotic effects on HuCCT-1 cells, which are mediated through control of CK2, caspase-9/3, DR-4, STAT-3/5, Mcl-1, eIF-2α, and HIF-1α.

## 1. Introduction

Cholangiocarcinoma (CCA), which forms in the bile ducts, currently accounts for ~15% of hepatobiliary cancers and ~3% of gastrointestinal malignancies [1,2], and men have a slightly higher incidence of CCA and mortality from cancer than women [3]. Although surgery, curative liver transplantation, and radiation therapy are options for patients with cholangiocarcinoma [4], the 5-year survival rate is around 40% or less even after curative surgery [4,5]. Thus, there remains a great need for more effective drugs to enhance locoregional disease control and overall survival in cholangiocarcinoma patients.

Casein kinase 2 (CK2) is a serine/threonine protein kinase that phosphorylates many intracellular protein substrates in normal and cancer cells. Accordingly, de-regulation of CK2 has been linked to tumorigenesis as a potential protection mechanism for cancer cells [6,7,8]. Of note, there is accumulating evidence that CK2 is overexpressed in CCA [9], and there is a significant association of CK2 overexpression with progression and prognosis of CCA [10]. The existing body of research on CK2 thus strongly suggests CK2 inhibition as a targeted therapy for CCA.

CX-4945 (Silmitasertib) is an inhibitor of CK2 with anti-cancerous and anti-angiogenic effects [11]. Of interest, it has been recently shown that CX-4945 is an effective treatment of CCA [12]. At present, however, the CX-4945 regulation of CCA is not fully understood. In this study, we investigated the inhibitory effect of CX-4945 on the growth of HuCCT-1 cells, a human CCA cell line. Here we report that CX-4945 has anti-growth, pro-apoptotic, and anti-angiogenic effects on HuCCT-1 cells, which are mediated through control of the expression, activation, and phosphorylation of CK2, caspase-9/3, death receptor-4 (DR-4), signal transducer and activator of transcription-3 (STAT-3), STAT-5, eukaryotic initiation factor-2α (eIF-2α), myeloid cell leukemia-1 (Mcl-1), and hypoxia-inducible factor 1α (HIF-1α).

## 2. Results

### 2.1. CX-4945 Inhibits Growth and Induces Apoptosis of HuCCT-1 Cells

**Figure 1:**
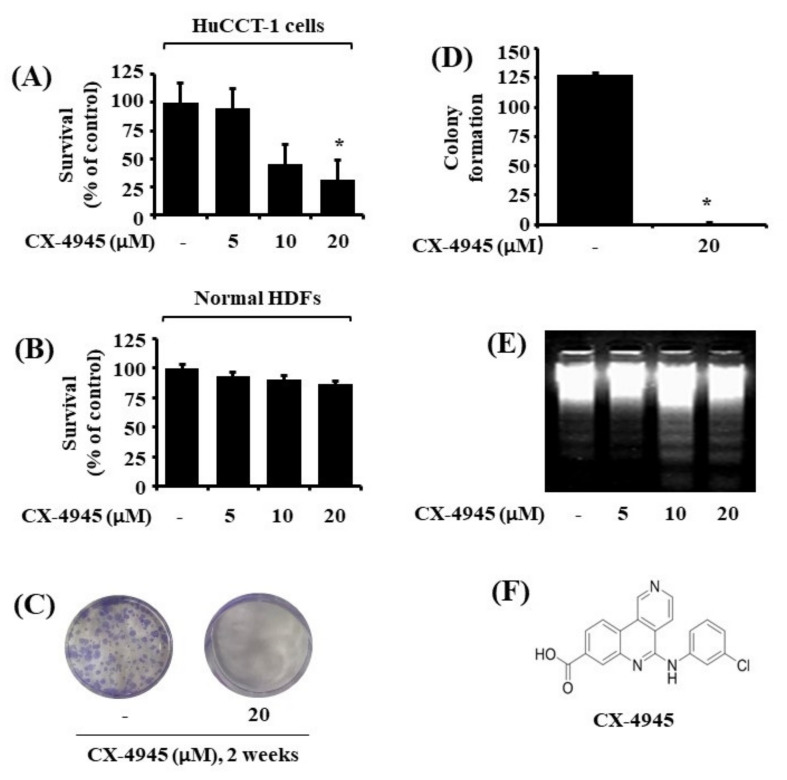
Effects of CX-4945 on cell growth (survival) and apoptosis (DNA fragmentation) on HuCCT-1 human cholangiocarcinoma cells. (**A**) HuCCT-1 cells were treated with CX-4945 or vehicle control (DMSO; 0.1%) at the indicated concentrations for 24 h. The number of surviving cells was measured by cell count assay. The cell count assay was performed in triplicate. Data are means ± SE of three independent experiments. * *p* < 0.05 compared to the value of CX-4945 or vehicle control (DMSO) for the indicated time. (**B**) Human dermal fibroblast (HDF) cells were treated with CX-4945 or vehicle control (DMSO; 0.1%) at the indicated concentrations for 24 h. The survival of surviving cells was measured by cell count assay. The cell count assay was performed in triplicate. Data are means ± SE of three independent experiments. (**C**) HuCCT-1 cells were treated with CX-4945 (20 µM) or vehicle control (DMSO) and incubated for 2 weeks, followed by crystal violet staining. Each image is representative of three independent experiments. (**D**) Densitometry analysis of (**C**). (**E**) HuCCT-1 cells were treated with CX-4945 or vehicle control (DMSO) at the indicated concentrations for 24 h. Extra-nuclear fragmented DNA was extracted and analyzed on a 1.7% agarose gel. (**F**) The chemical structure of CX-4945.

We initially investigated the effect of CX-4945 at different concentrations (5, 10, and 20 µM) for 24 h on the survival of HuCCT-1 cells by using cell count analysis. As shown in Figure 1A, CX-4945 induced a concentration-dependent reduction of HuCCT-1 cell survival compared with control cells. To know whether the CX-4945’s anti-survival effect is only limited to HuCCT-1 cells, we additionally examined the effect of CX-4945 at different concentrations on the growth of SNU-1196, another human CCA cell line. Similarly, data from cell count assay revealed the ability of CX-4945 to reduce the survival of SNU-1196 cells in a dose-dependent fashion (Supplementary Figure S1A). The CX-4945’s growth-suppressive effect on SNU1196 cells was also confirmed by a phase-contrast microscope (Supplementary Figure S1B). To further see the specificity, we next tested the effect of CX-4945 at 5, 10, and 20 µM for 24 h on the survival of normal HDFs. As shown in Figure 1B, CX-4945 at the doses tested was not cytotoxic to HDFs. Using a clonogenic assay, we next sought to explore whether CX-4945 inhibits the survival and proliferation of HuCCT-1 cells. As shown in Figure 1C, there was a markedly diminished colony formation of HuCCT-1 cells treated with CX-4945 at 20 μM for 2 weeks. Densitometric data of Figure 1C is shown as Figure 1D. Next, whether CX-4945 induces apoptosis of HuCCT-1 cells was determined by measuring nuclear DNA fragmentation, a hallmark of apoptosis. As shown in Figure 1D, CX-4945 treatment at 10 or 20 µM for 24 h resulted in an apparent nuclear DNA fragmentation in HuCCT-1 cells. The chemical structure of CX-4945 is shown in Figure 1E. These results demonstrated that CX-4945 at 20 µM has solid anti-survival and pro-apoptotic effects on HuCCT-1 cells. Because of its strong growth-suppressive and apoptosis-inducing effects on HuCCT-1 cells, this 20 µM concentration of CX-4945 was chosen for further studies.

### 2.2. CX-4945 at 20 µM Induces PARP Cleavage, Activation of Caspase-9/3, and Up-Regulation of DR-4 in HuCCT-1 Cells

**Figure 2:**
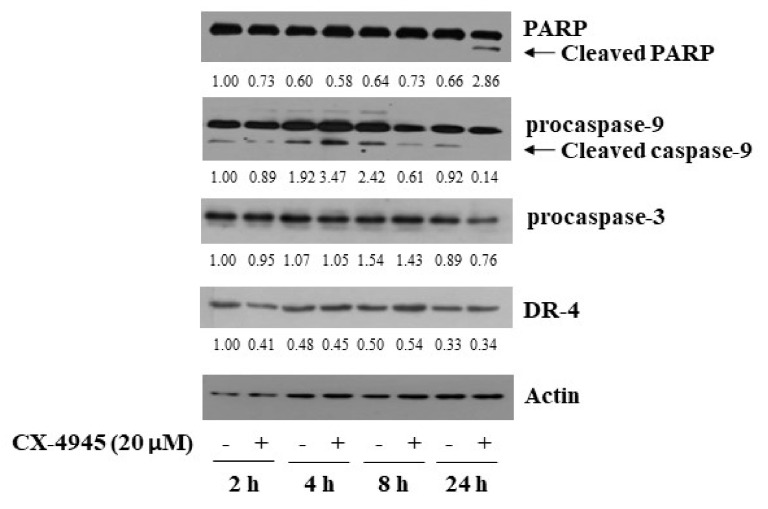
Effects of CX-4945 on the expression of PARP, procaspase-9/3 and DR-4 in HuCCT-1 cells. HuCCT-1 cells were treated with CX-4945 (20 μM) or vehicle control (DMSO) for the time designated. At each time points, whole cell lysates were prepared and analysed for levels of PARP, procaspase-9, procaspase-3, DR-4 or β-actin by Western Blotting.

To understand molecular mechanisms by which CX-4945 inhibits growth and induces apoptosis of HuCCT-1 cells, we next carried out time course experiments to know whether CX-4945 modulates the expression of apoptosis-related proteins, including PARP, caspases, and death receptors (DRs), in HuCCT-1 cells. In this study, the ability of CX-4945 to induce activation of caspase-9/3 in HuCCT-1 cells was assessed by measuring not only decreased expression levels of procaspase-9/3 but increased expression levels of cleaved (active) caspase-9/3 but also generation of cleaved PARP, which is mediated by active caspases. As shown in Figure 2, treatment with CX-4945 at 24 h resulted in high levels of cleaved PARP in HuCCT-1 cells compared with control cells. Moreover, CX-4945 treatment led to increased expression levels of cleaved caspase-9 at 4 h while decreasing expression levels of procaspase-3 at 24 h in HuCCT-1 cells. In addition, there was a slight elevation of DR-4 protein expression in HuCCT-1 cells treated with CX-4945 at 8 and 24 h. Expression levels of control actin protein remained constant under these experimental conditions.

### 2.3. CX-4945 at 20 µM Vastly Reduces Phosphorylation Levels of CK2 Substrates and Knockdown of CK2 Leads to a Partial Reduction of HuCCT-1 Cell Survival

**Figure 3:**
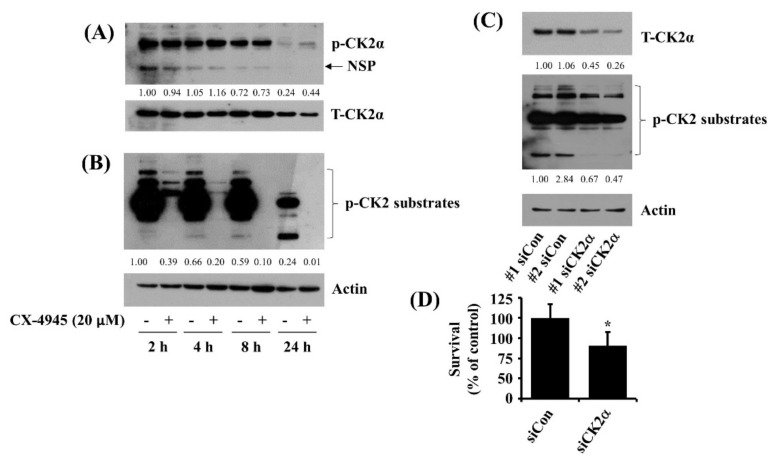
Effect of CX-4945 on phosphorylation and expression levels of CK2α and CK2 substrates in HuCCT-1 cells. (**A**,**B**) HuCCT-1 cells were treated with CX-4945(20 μM) or vehicle control (DMSO) for the time designated. At each time point, whole-cell lysates were prepared and analyzed for levels of p-CK2α, T-CK2α, p-CK2 substrates, or β-actin by Western Blotting. (**C**) HuCCT-1 cells were transfected with 200 pM of control siRNA(siCon) or CK2α siRNA (siCK2α) for 48 h. Whole-cell lysates were prepared and analyzed for T-CK2α, p-CK2 substrates, or β-actin by Western Blotting. (**D**) HuCCT-1 cells were transfected with 200 pM of control siRNA (siCon) or CK2α siRNA (siCK2α) for 48 h, then measured the number of surviving cells by cell count assay. The cell count assay was performed in triplicate. Data are means ± SE of three independent experiments. * *p* < 0.05 compared to the value of siCon or siCK2α at the indicated time.

CX-4945 is a pharmacological inhibitor of CK2α [11]. Targeting CK2 has been proposed for the treatment of CCA [13]. Given that CK2 is a constitutively expressed and active Ser/Thr protein kinase that phosphorylates hundreds of protein substrates, we next asked whether CK2α is expressed and phosphorylated and has kinase activity in HuCCT-1 cells. As shown in Figure 3A, in the absence of CX-4945, results of the kinetic study demonstrated that there were substantial levels of phosphorylated CK2α at 2, 4, and 8 h, followed by a significant decline at 24 h (upper panel). There were also high total expression levels of CK2α at 2, 4, and 8 h, followed by a substantial reduction at 24 h (lower panel). Furthermore, as shown in Figure 3B, of interest, there were high levels of phosphorylated CK2 substrates at 2, 4, and 8 h, followed by a big decrease at 24 h (upper panel). As expected, CX-4945 did not alter the expression and phosphorylation levels of CK2α in HuCCT-1 cells (Figure 3A). Still, the drug vastly reduced levels of phosphorylated CK2 substrates in HuCCT-1 cells at times tested (Figure 3B). These results point out that CK2α is constitutively expressed/phosphorylated and has the kinase activity in HuCCT-1 cells, and CX-4945 strongly inhibits CK2α activity and its downstream pathway without affecting its protein expression levels in the cells. Control actin protein levels remained unchanged under these experimental conditions. We next sought to explore the role of reduced CK2α activity in the CX-4945’s anti-survival effect on HuCCT-1 cells by using CK2α siRNA transfection. As shown in Figure 3C, there were fewer protein expression levels of CK2α in the CK2α siRNA-transfected HuCCT-1 cells than those in control siRNA-transfected ones (upper panel). In addition, there were fewer levels of phosphorylated CK2 substrates in CK2α siRNA-transfected HuCCT-1 cells than those in control siRNA-transfected ones (middle panel). Control actin protein levels remained constant under these experimental conditions (lower panel). Importantly, as shown in Figure 3D, data of cell count analysis showed that knockdown of CK2α led to a significant reduction of HuCCT-1 cell survival.

### 2.4. CX-4945 at 20 µM Reduces Phosphorylation and Expression Levels of STAT-3 and STAT-5 in HuCCT-1 Cells

**Figure 4:**
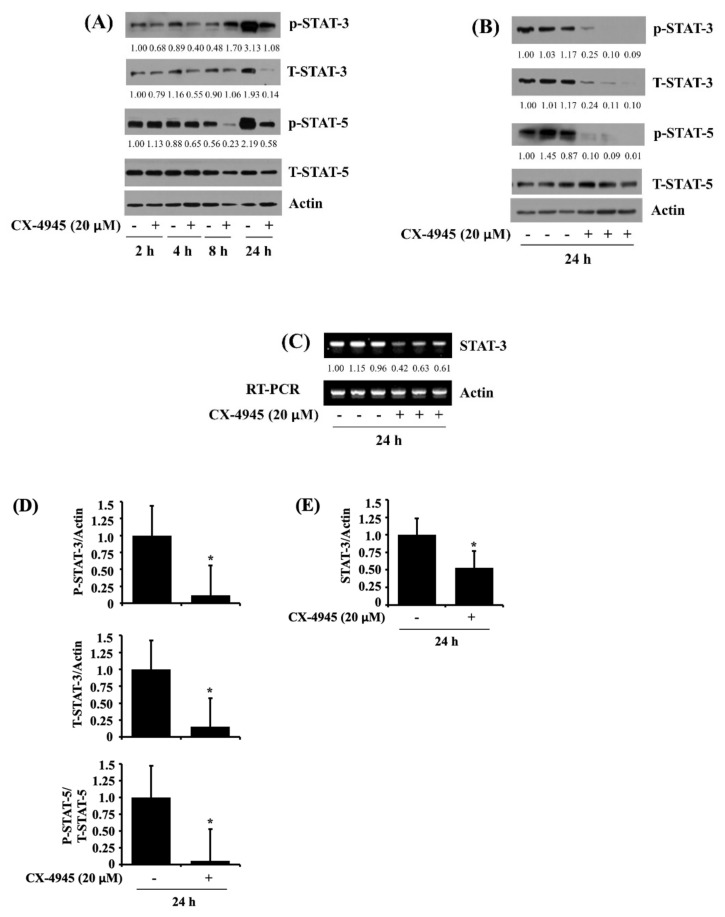
Effect of CX-4945 on expression and phosphorylation levels of STAT-3 and STAT-5 in HuCCT-1 cells. (**A**) HuCCT-1 cells were treated with CX-4945 (20 μM) or vehicle control (DMSO) at indicated time points. At each time point, whole-cell lysates were prepared and analyzed for levels of p-STAT-3, T-STAT-3, p-STAT-5, T-STAT-5, or β-actin by Western blotting. (**B**) HuCCT-1 cells were treated with CX-4945 (20 μM) or vehicle control (DMSO) in triplicate experiments at 24 h. Then whole-cell lysates were prepared and analyzed for levels of p-STAT-3, T-STAT-3, p-STAT-5, T-STAT-5, or β-actin by Western blotting. (**C**) HuCCT-1 cells were treated with CX-4945 (20 μM) or vehicle control (DMSO) in triplicate experiments at 24 h. Total cellular RNA was extracted and analyzed for levels of STAT-3 or β-actin by RT-PCR with respective primers. (**D**) Densitometry analysis of (**B**). Data are mean ± SE of three independent experiments. * *p* < 0.05 compared to vehicle control. (**E**) Densitometry analysis of (**C**). Data are mean ± SE of three independent experiments. * *p* < 0.05 compared to vehicle control.

Evidence suggests a role of STATs in cancer cell survival [14,15] and apoptosis [16]. To date, little is known about the expression and phosphorylation (activation) of STATs in CCA cells. We thus checked whether STAT-3 and STAT-5, two critical members of the STATs family, are expressed and phosphorylated in HuCCT-1 cells. Notably, as shown in Figure 4A, there were substantial expression and phosphorylation levels of STAT-3 and STAT-5 in HuCCT-1 cells at times tested. Maximal phosphorylation levels of STAT-3 and STAT-5 were seen at 24 h. However, CX-4945 treatment, particularly at 24 h, led to a substantial reduction of the phosphorylation and expression levels of STAT-3 protein in HuCCT-1 cells. In addition, CX-4945 treatment at 24 h strongly down-regulated STAT-5 protein phosphorylation levels, but it slightly reduced the protein expression levels in HuCCT-1 cells. Results of Western blotting from triplicate experiments, as shown in Figure 4B, further confirmed the ability of CX-4945 to significantly inhibit not only the phosphorylation and expression of STAT-3 but also the phosphorylation of STAT-5 in HuCCT-1 cells. Using the RT-PCR experiment, we further tested whether the reduced STAT-3 protein expression by CX-4945 was due to decreased STAT-3 transcripts in HuCCT-1 cells. As shown in Figure 4C, data of RT-PCR analysis from triplicate experiments demonstrated that CX-4945 significantly down-regulates STAT-3 mRNA expression in HuCCT-1 cells. Densitometric data of Figure 4B,C for the protein phosphorylation and expression levels of STAT-3 and STAT-5 normalized to those of control actin or total STAT-5, and the mRNA expression levels of STAT-3 normalized to those of control actin are shown in Figure 4D,E respectively.

### 2.5. Pharmacological Inhibition or Respective Knockdown of STAT-3 and STAT-5 Leads to Reduction of HuCCT-1 Cell Survival

**Figure 5:**
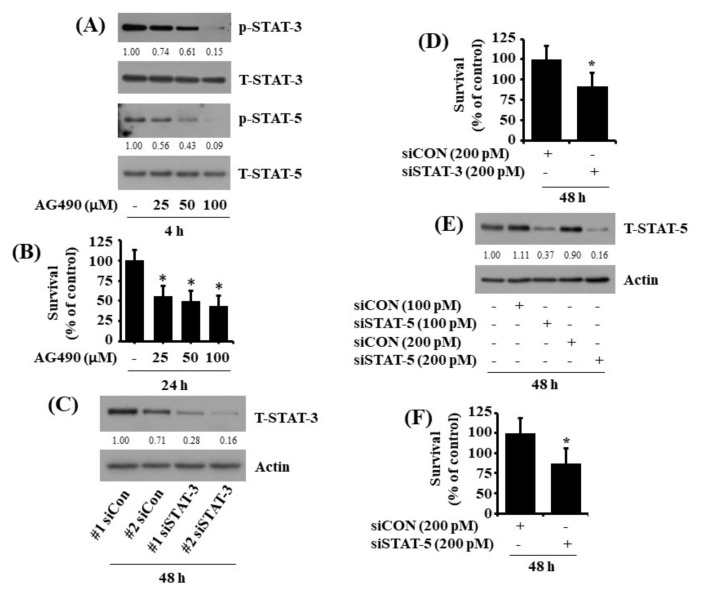
Effects of AG490 or STAT-3 or STAT-5 on the expression and phosphorylation of STAT-3 and STAT-5 in HuCCT-1 cells. (**A**) HuCCT-1 cells were treated with AG490 at the indicated concentrations for 4 h. Whole-cell lysates were prepared and analysed for levels of p-STAT-3, T-STAT-3, p-STAT-5, T-STAT-5, or β-actin by Western blotting. (**B**) HuCCT-1 cells were treated with AG490, a pan-inhibitor of STATs, at the indicated concentrations for 24 h, then measured the number of surviving cells by cell count assay. The cell count assay was performed in triplicate. Data are means ± SE of three independent experiments. * *p* < 0.05 compared to the control at the indicated concentrations. (**C**) HuCCT-1 cells were transfected with 200 pM of control siRNA (siCon) or STAT-3 siRNA (siSTAT-3) for 48 h. Whole-cell lysates were prepared and analyzed for T-STAT-3 or β-actin by Western Blotting. (**D**) HuCCT-1 cells were transfected with 200 pM of control siRNA (siCon) or STAT-3 siRNA (siSTAT-3) for 48 h, followed by measurement of the number of surviving cells by cell count assay. The cell count assay was performed in triplicate. Data are means ± SE of three independent experiments. * *p* < 0.05 compared to the value of siCon or siSTAT-3 at the indicated time. (**E**) HuCCT-1 cells were transfected with 200 pM of control siRNA (siCon) or STAT-5 siRNA (siSTAT-5) for 48 h. Whole-cell lysates were prepared and analyzed for T-STAT-5 or β-actin by Western Blotting. (**F**) HuCCT-1 cells were transfected with 200 pM of control siRNA (siCon) or STAT-5 siRNA (siSTAT-5) for 48 h, followed by measurement of the number of surviving cells by cell count assay. The cell count assay was performed in triplicate. Data are means ± SE of three independent experiments. * *p* < 0.05 compared to the value of siCon or siSTAT-5 at the indicated time.

Using AG490, a pan-inhibitor of STATs, we next investigated the role of reduced expression and activity of STAT-3 and STAT-5 in the CX-4945-induced growth suppression of HuCCT-1 cells. As shown in Figure 5A, treatment with AG490 for 4 h concentration-dependently inhibited the phosphorylation of STAT-3 and STAT-5 without affecting their total expression levels in HuCCT-1 cells, pointing out the drug efficacy. Notably, AG490 treatment for 24 h further resulted in a significant reduction of HuCCT-1 cell survival in a dose-dependent manner (Figure 5B). We next performed a siRNA transfection experiment to directly see the role of STAT-3 or STAT-5 in HuCCT-1 cell survival. Results of gene silencing demonstrated that compared with control siRNA-transfected HuCCT-1 cells, there were much fewer expression levels of STAT-3 or STAT-5 in STAT-3 or STAT-5 siRNA-transfected cells (Figure 5C,E), and knockdown of STAT-3 or STAT-5 caused a significant reduction of HuCCT-1 cell survival (Figure 5D,F).

### 2.6. CX-4945 at 20 µM Reduces Protein Expression of Mcl-1 and Elevates Protein Phosphorylation of eIF-2α in HuCCT-1 Cells

**Figure 6:**
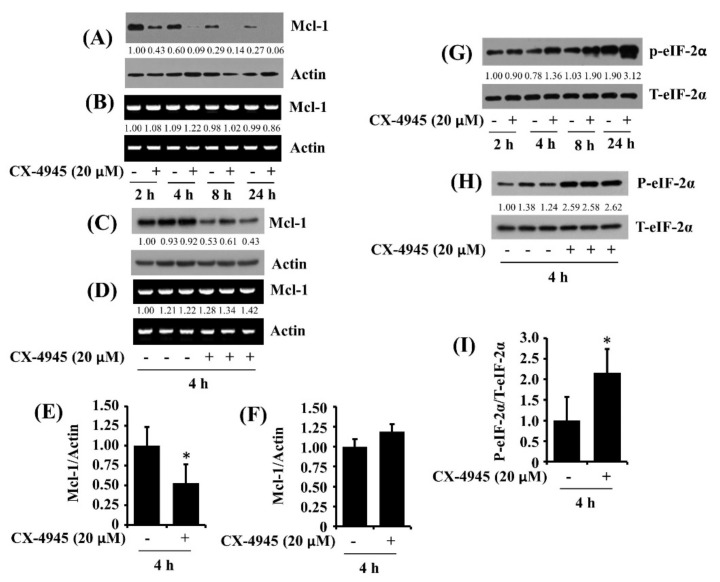
Effects of CX-4945 on expression and phosphorylation of Mcl-1, eIF-2α in HuCCT-1 cell. (**A**) HuCCT-1 cells were treated with CX-4945 (20 μM) or vehicle control (DMSO) at indicated time points. At each time point, whole-cell lysates were prepared and analyzed for levels of Mcl-1 or β-actin by Western blotting. (**B**) HuCCT-1 cells were treated with CX-4945 (20 μM) or vehicle control (DMSO) at indicated time points. At each time point, total cellular RNA was abstracted and analyzed for levels of Mcl-1 or β-actin by RT-PCR. (**C**) Western blotting analysis in triplicate experiments for 4 h. (**D**) RT-PCR analysis in triplicate experiments for 4 h. (**E**) The densitometry data of (**C**). * *p* < 0.05 compared to the control at the indicated time. (**F**) The densitometry data of (**D**). * *p* < 0.05 compared to the control at the indicated time. (**G**) HuCCT-1 cells were treated with CX-4945 (20 μM) or vehicle control (DMSO) at indicated time points. At each time point, whole-cell lysates were prepared and analyzed for levels of p-eIF-2α and T-eIF-2α by Western blotting. (**H**) Western blotting analysis in triplicate experiments for 4 h. (**I**) The densitometry data of (**H**). * *p* < 0.05 compared to the control at the indicated time.

We next examined whether CX-4945 affects the expression of the Bcl-2 family, anti-apoptotic proteins [17], in HuCCT-1 cells. Interestingly, as shown in Figure 6A, data of time course works showed high protein expression levels of Mcl-1 at 2 h but a sharp decline of the protein levels after that in HuCCT-1 cells. Of note, treatment with CX-4945 led to a substantial reduction of the protein expression levels of Mcl-1 in HuCCT-1 cells at times tested. Control actin protein levels remained constant under these experimental conditions. We next sought to explore whether Mcl-1 protein down-regulation by CX-4945 was due to reducing Mcl-1 transcripts in HuCCT-1 cells. Distinctly, CX-4945 treatment did not affect Mcl-1 mRNA expression in HuCCT-1 cells at times tested (Figure 6B). Results of Western blot and RT-PCR analysis from triplicate experiments, as shown in Figure 6C,D, further revealed the ability of CX-4945 to significantly inhibit the protein expression of Mcl-1 with no change in Mcl-1 mRNA expression in HuCCT-1 cells. Densitometric data of Figure 6C for the protein expression levels and Figure 6D for mRNA expression levels of Mcl-1 normalized to those of control actin are shown in Figure 6E,F. We then examined whether CX-4945 affects the expression and phosphorylation of eIF-2α, a translation-related protein [18], in HuCCT-1 cells. As shown in Figure 6G, there was a time-dependent increase in levels of phosphorylated eIF-2α protein in HuCCT-1 cells. Maximal phosphorylation of eIF-2α was seen at 24 h. However, of interest, treatment with CX-4945 further augmented levels of phosphorylated eIF-2α without influencing the protein expression levels in HuCCT-1 cells at times tested. Results of Western blot analysis from triplicate experiments, as shown in Figure 6H, further demonstrated the ability of CX-4945 to significantly elevate the phosphorylation of eIF-2α without altering the protein expression levels in these cells. Densitometric data of Figure 6H for the phosphorylation levels of eIF-2α normalized to those of the protein’s total expression levels is shown in Figure 6I.

### 2.7. CX-4945-Induced Decrease in the Expression and Phosphorylation of STAT-3/-5 Is the CK-2-Independent but CX-4945-Induced Increase in eIF-2α Phosphorylation Is the CK-2-Dependent in HuCCT-1 Cells

**Figure 7:**
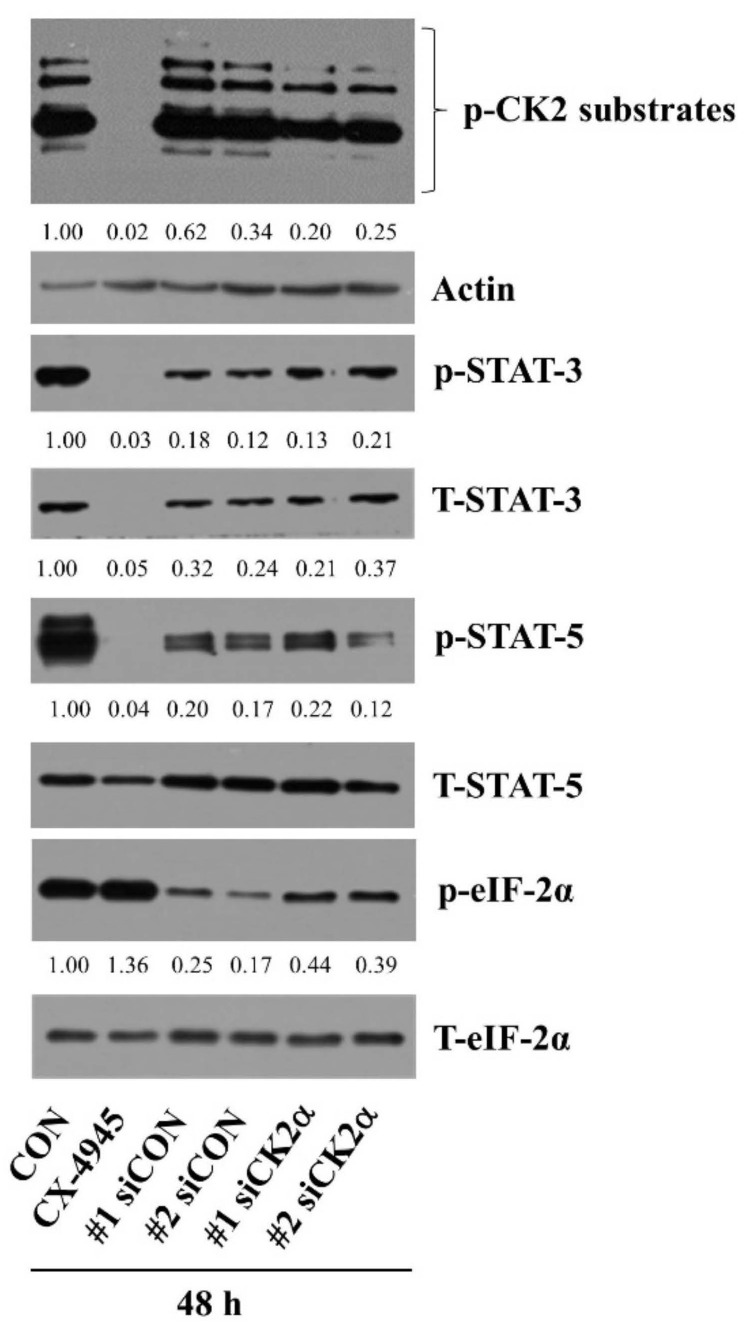
Effects of CX-4945 and CK-2α siRNA on the expression and phosphorylation of STAT-3, STAT-5, and eIF-2α in HuCCT-1 cells. HuCCT-1 cells were treated with CX-4945 (20 μM) or vehicle control (DMSO) or transfected with 200 pM of control siRNA (siCon) or CK-2 siRNA (siCK2α) for 48 h. Whole-cell lysates were prepared and analyzed for levels of p-CK-2 substrates, p-STAT-3, T-STAT-3, p-STAT-5, T-STAT-5, p-eIF-2α, T-eIF-2α, or β-actin by Western blotting.

Using gene silencing of CK2, we next sought to explore whether CX-4945-induced alteration of the expression and phosphorylation of STAT-3, STAT-5, and eIF-2α in HuCCT-1 cells is the CK2-dependent or not. As shown in Figure 7, compared with control cells (no CX-4945), treatment with CX-4945 for 48 h led to a decrease in the phosphorylation and expression of CK2 substrates, STAT-3, and STAT-5 in HuCCT-1 cells, but it caused a slight elevation of eIF-2α phosphorylation in these cells. As anticipated, there was a decrease in phosphorylated CK2 substrates in CK2α siRNA-transfected HuCCT-1 cells compared with those in control siRNA-transfected ones. However, there was no reduction of the phosphorylation and expression levels of STAT-3 and STAT-5 in CK2α siRNA-transfected HuCCT-1 cells compared with those in control siRNA-transfected ones. Rather there was a slight increase in their phosphorylation and expression levels. Notably, there were higher levels of phosphorylated eIF-2α in CK2α siRNA-transfected HuCCT-1 cells than those in control siRNA-transfected ones. Total expression levels of eIF-2α remained constant under these experimental conditions.

### 2.8. CX-4945 at 20 µM Down-Regulates HIF-1α in HuCCT-1 Cells Time-Differentially and Knockdown of HIF-1α Leads to a Significant Reduction of the Cell Survival

**Figure 8:**
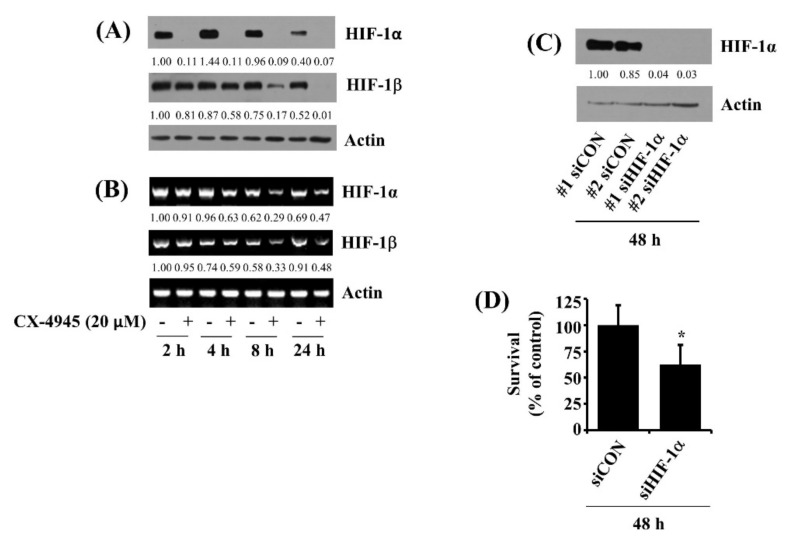
Effects of CX-4945 on the expression of HIF-1α in HuCCT-1 cells. (**A**) HuCCT-1 cells were treated with CX-4945 (20 μM) or vehicle control (DMSO) at indicated time point. At each time point, whole-cell lysates were prepared and analyzed for levels of HIF-1α, HIF-1β, or β-actin by Western blotting. (**B**) HuCCT-1 cells were treated with CX-4945 (20 μM) or vehicle control (DMSO) at indicated time points. At each time point, total cellular RNA was abstracted and analyzed for levels of HIF-1α, HIF-1β, or β-actin by RT-PCR. (**C**) HuCCT-1 cells were transfected with 200 pM of control siRNA (siCon) or HIF-1α siRNA (siHIF-1α) for 48 h. Whole-cell lysates were prepared and analyzed for HIF-1α, HIF-1β, or β-actin by Western Blotting. (**D**) HuCCT-1 cells were transfected with 200 pM of control siRNA (siCon) or HIF-1α siRNA (siHIF-1α) for 48 h, followed by measurement of the number of surviving cells by cell count assay. The cell count assay was performed in triplicate. Data are means ± SE of three independent experiments. * *p* < 0.05 compared to the value of siCon or siHIF-1α at the indicated time.

HIF-1α is an angiogenic transcription factor [19], and its overexpression is partially linked to CCA survival and metastasis [20]. This led us to test whether HIF-1α is expressed in HuCCT-1 cells and whether CX-4945 regulates it. As shown in Figure 8A, results of time course work illustrated sustained protein expression levels of HIF-1α in HuCCT-1 cells at times tested. Similarly, there were also sustained protein expression levels of HIF-1β in these cells for the times applied. Strikingly, treatment with CX-4945 at 2 h caused a complete loss of HIF-1α protein with no change of HIF-1β protein in HuCCT-1 cells. However, CX-4945 treatment at 4, 8, and 24 h resulted in a time-dependent reduction of HIF-1α and HIF-1β proteins in HuCCT-1 cells. Control actin protein levels remained constant under these experimental conditions (Figure 8A). Strikingly, as shown in Figure 8B, data of RT-PCR analysis treatment with CX-4945 at 2 h did not affect the mRNA expression levels of HIF-1α and HIF-1β in HuCCT-1 cells. However, CX-4945 treatment at 4 h and after that also led to a time-dependent down-regulation of HIF-1α and HIF-1β transcripts in these cells. Using HIF-1α siRNA transfection, we next sought to explore the role of HIF-1α down-regulation in the CX-4945′s anti-survival effect on HuCCT-1 cells. As shown in Figure 8C, there was a complete loss of HIF-1α in HIF-1α siRNA-transfected HuCCT-1 cells compared with that in control siRNA-transfected ones. Control actin protein levels remained unchanged under these experimental conditions. Of importance, data of cell count analysis demonstrated that knockdown of HIF-1α led to a significant reduction of HuCCT-1 cell survival (Figure 8D).

## 3. Discussion

CK2 is a constitutively expressed and active protein kinase with a long history as a pro-survival and anti-apoptotic kinase. Given the widespread overexpression of CK2 in multiple cancers, a selective inhibitor of CK2 is an attractive targeted approach to treating cancer. Although several inhibitors of CK2 have been discovered in the last 20 years, only CX-4945 has entered into clinical trials as a potential anti-cancer drug. Notably, preclinical in vitro and in vivo evidence of an anti-cancer effect of CX-4945 alone or the combination of gemcitabine and/or cisplatin in CCA has been reported [12,21]. However, up to date, the anti-cancer effect and mode of action of CX-4945 in CCA are not fully understood. Here we show that CX-4945 has anti-survival, pro-apoptotic, and anti-angiogenic effects, and these effects are mediated through control of multiple targets, including CK2, caspase-9/3, DR-4, STAT-3/5, eIF-2α, Mcl-1, and HIF-1α.

It is known that CX-4945 has anti-tumor activity against many human solid cancer cells, such as gastric [22], breast [23], pancreatic [24], and hematologic [25]. Of interest, it has been recently shown that CX-4945 has an anti-proliferative effect on HuCCT-1 cells and the apoptosis-inducing effect on mouse xenograft model via regulation of CK2, caspase-3/7, PKB, and DNA-repairing enzymes [12]. We also have demonstrated that CX-4945 (10 or 20 μM) strongly inhibits the growth of HuCCT-1 cells, but it is not cytotoxic to normal cells, addressing the drug’s selectivity to inhibit CCA cells. Cancer cells undergoing apoptosis have several distinct biochemical characteristics, including nuclear DNA fragmentation and cleavage of PARP [26]. Thus, assuming the present findings that CX-4945 induces nuclear DNA fragmentation and PARP cleavage in HuCCT-1 cells, it is evident that this CK2 inhibitor also induces apoptosis of HuCCT-1 cells. Reportedly, apoptosis induction is mainly initiated from different entry points, for example, at the plasma membrane upon ligation of DRs (extrinsic pathway) or the mitochondria (intrinsic pathway) [26]. Given that CX-4945 increases expression levels of cleaved caspase-9 while decreasing those of procaspase-3 and it concomitantly up-regulates DR-4 in HuCCT-1 cells, it is likely that the CX-4945-induced apoptosis of HuCCT-1 cells is dependent on both the caspase-dependent intrinsic pathway and the DR-mediated extrinsic pathway.

CK2 is a tetrameric enzyme composed of 2 catalytic (α and or α’) subunits and 2 regulatory (β) subunits [27]. CK2 is reported to phosphorylate a hundred protein substrates in cells and play a crucial role in cancer cells’ proliferation, survival, and malignant phenotype, including CCA [10,28]. Although CK2β expression in CCA cells and its survival role have been previously reported [29], little is known about the expression and phosphorylation and function of CK2α in CCA cells. In the current study, we have demonstrated that CK2α is expressed and phosphorylated, and has kinase activity, as evidenced by an elevated level of phosphorylated CK2 substrates in HuCCT-1 cells. In the current study, CX-4945 does not alter the expression and phosphorylation of CK2α. Still, it vastly lowers levels of phosphorylated CK2 substrates in HuCCT-1 cells, pointing out the drug’s ability to selectively inhibit the kinase activity of CK2. Importantly, results of gene silencing herein have revealed that knockdown of CK2α that substantially decreases levels of phosphorylated CK2 substrates in HuCCT-1 cells further leads to a significant reduction of cell survival. These results strongly suggest that CK2α is a survival factor in HuCCT-1 cells. and the CX-4945′s anti-survival effect on HuCCT-1 cells is mediated through inhibition of CK2α and its downstream signaling pathway(s).

The transcription factor STAT-3 is overexpressed and plays oncogenic roles in many cancers [30]. It is further noted that STAT-3 contributes to CCA carcinogenesis and progression and may serve as a marker for a poor prognosis of CCA [31]. This study shows that the expression and phosphorylation of STAT-3 and STAT-5, another member of the STATs family, are detected in HuCCT-1 cells. Until now, CX-4945 regulation of STAT-3 and STAT-5 in HuCCT-1 cells and the role of STAT-3 and STAT-5 are not fully defined. Distinctly, we have demonstrated that CX-4945 inhibits both the phosphorylation and expression of STAT-3, but it blocks only the phosphorylation of STAT-5 in HuCCT-1 cells. These results indicate that CX-4945 inhibits both STAT-3 and STAT-5 in HuCCT-1 cells. The drug’s inhibitory effect on STAT-3 and STAT-5 is due to the former’s transcriptional repression and the latter’s dephosphorylation at the protein (post-translational) level, respectively. Further, assuming the present results that pharmacological inhibition or respective knockdown of STAT-3 and STAT-5 in HuCCT-1 cells leads to a significant reduction of HuCCT-1 cell growth, it is likely that inhibition of STAT-3 and STAT-5 by CX-4945 contributes to the drug’s anti-survival effect on these cells. Previously, CK2 regulation of STAT-3 in hematological malignances has been reported [32,33]. However, data of gene silencing of CK2α herein illustrates that knockdown of CK2α does not influence the phosphorylation and expression of STAT-3 and STAT-5 in HuCCT-1 cells. These results indicate that CK2α does not lie upstream of STAT-3 and STAT-5 in HuCCT-1 cells, and the CX-4945-induced inhibition of STAT-3 and STAT-5 in these cells is mediated not through inhibition of CK2α but regulation of another factor(s) or pathway(s). It will be interesting to examine, in the future, which kinase(s) or factor(s) regulate the phosphorylation and expression of STAT-3 and STAT-5 in HuCCT-1 cells in response to CX-4945 exposure by using kinomics or RNA sequencing approach.

Mcl-1 is an anti-apoptotic protein of the Bcl-2 family [34]. Overexpression of Mcl-1 is frequently detected in many tumors and is closely associated with tumorigenesis, poor prognosis, and drug resistance [35]. In this study, Mcl-1 is expressed in HuCCT-1 cells, and CX-4945 vastly down-regulates Mcl-1 at levels of protein, but not mRNA. These results point out that CX-4945-induced Mcl-1 down-regulation in HuCCT-1 cells is due to decreased protein synthesis or increased protein turnover. It is thus likely that the loss of Mcl-1 may further contribute to the drug’s anti-survival and/or pro-apoptotic effects. In this study, we also have hypothesized that CX-4945 might exert its anti-survival and pro-apoptotic effects through the regulation of additional pathways (components). A wealth of information indicates that many anti-cancer drugs or agents induce ER stress and/or inhibit protein synthesis (translation) in cancer cells, which are crucial for their anti-survival and/or pro-apoptotic effects. eIF-2α, glucose-regulated protein 78 (GRP78), and activating transcription factor 4 (ATF4) are known ER stress and translation-related markers. We thus have investigated in this study to see whether CX-4945 alters the expression and phosphorylation levels of eIF-2α, GRP78, and ATF4 in HuCCT-1 cells. Distinctly, the present study has revealed that CX-4945 elevates eIF-2α phosphorylation but has no effects on expression levels of GRP78 and ATF4 (Supplementary Figure S2) in HuCCT-1 cells. eIF-2α is a protein that controls the translation, and thus its expression and (de)phosphorylation status greatly influence cancer cell growth and survival [36]. It is documented that the non-phosphorylated eIF-2α is active while the phosphorylated one is inactive in the translation initiation complex formation. Accordingly, eIF-2α hyperphosphorylation in response to environmental stress or drug exposure reduces global translation [37]. Of note, previous studies have demonstrated the CX-4945-induced eIF-2α hyperphosphorylation in eye cells [38] and leukemia cells [39]. In agreement with this, we have shown the capability of CX-4945 to elevate the phosphorylation of eIF-2α in HuCCT-1 cells. These results may thus imply that CX-4945 elicits eIF-2α hyperphosphorylation-dependent translational inhibition in HuCCT-1 cells, which may facilitate the drug’s cytotoxic effects. Given that knockdown of CK2α also leads to increased eIF-2α phosphorylation in HuCCT-1 cells in this study, it is further speculative that CK2α is a protein kinase that phosphorylates eIF-2α in these cells in response to CX-4945 exposure.

HIF-1 is a heterodimer composed of HIF-1α and HIF-1β subunits, and because of its crucial role in tumor angiogenesis, it has been recognized as an important cancer drug target [40]. Indeed, numerous studies demonstrate a strong correlation between HIF-1 overexpression and tumor metastasis, angiogenesis, and cancer resistance therapy [41]. Of interest, there is evidence that HIF-1α is expressed and plays a vital role in CCA [42]. At present, the expression and regulation of HIF-1α in CCA remain unclear. In addition, the CX-4945 regulation of HIF-1α in CCA is not fully understood. In this study, we have observed that HuCCT-1 cells express both HIF-1α and HIF-1β. Strikingly, while the short-term (2 h) treatment with CX-4945 only down-regulates HIF-1α at the protein, but not mRNA, levels, the long-term (4 to 24 h) treatment with this CK2 inhibitor results in a marked down-regulation of both HIF-1α and HIF-1β at their protein and mRNA levels. These results point out that CX-4945 time-differentially down-regulates HIF-1α in HuCCT-1 cells and the short-term administration of this drug may be effective in achieving a significant loss of HIF-1α at the protein levels. Given that HIF-1α is a tumor angiogenic factor, the present study suggests that CX-4945 may have a potential anti-angiogenic effect in HuCCT-1 cells. However, the only evidence of a possible anti-angiogenic effect of CX-4945 found in this study is the down-regulation of HIF-1α. Thus, to claim the anti-angiogenic result of CX-4945, it will be necessary for the future to examine whether CX-4945 can inhibit tube formation in the phorbol-12-myristate-13-acetate-treated human umbilical vascular endothelial cells, a well-established functional assay to claim the drug’s anti-angiogenic effect in culture. Further, considering that knockdown of HIF-1α leads to a significant reduction of HuCCT-1 cell survival herein, it is likely that HIF-1α may act as a survival factor in HuCCT-1 cells. Thus, it appears that the loss of HIF-1α may further contribute to the CX-4945′s anti-survival effect on HuCCT-1 cells.

**Figure 9:**
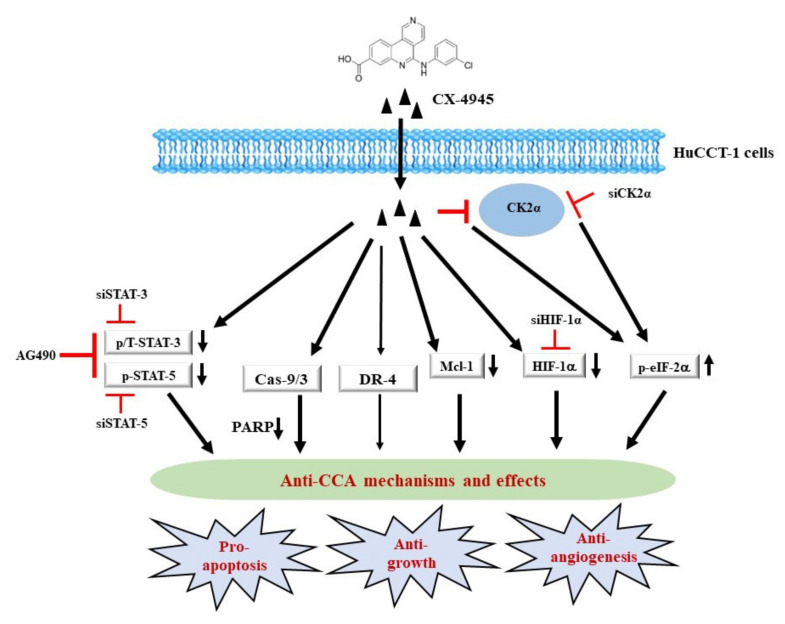
A diagram of the CX-4945’s anti-cancer mechanisms in HuCCT-1 CCA cells. CX-4945 may directly inhibit its target CK2 and downstream effector pathways. CX-4945 may further indirectly regulate additional targets, including the intrinsic (caspase-9/3) and extrinsic (DR-4) pathways, STAT-3/5, Mcl-1, and HIF-1α. Moreover, CX-4945 may induce eIF-2α-dependent global translational inhibition. These alterations will contribute to the drug’s anti-survival, pro-apoptotic, and anti-angiogenic effects on HuCCT-1 cells.

It is proposed that a likely scenario of possible molecular and cellular mechanisms underlying the CX-4945′s anti-survival and pro-apoptotic effects on HuCCT-1 cells herein is that (1) CX-4945 may directly inhibit its target CK2 and downstream effector pathways (components), (2) CX-4945 may also indirectly regulate additional targets, including activation of the intrinsic (caspase-9/3) and extrinsic (DR-4) pathways, inhibition of phosphoproteins (STAT-3/5), down-regulation of an anti-apoptotic protein (Mcl-1), suppression of a tumor angiogenic transcription factor (HIF-1α), and (3) CX-4945 may induce global translation inhibition (eIF-2α hyperphosphorylation), which all contribute to the drug’s anti-survival, pro-apoptotic, and possible anti-angiogenic effects on HuCCT-1 cells (Figure 9).

In summary, we demonstrate that CX-4945 has anti-growth, anti-angiogenic, and pro-apoptotic effects on HuCCT-1 cells, mediated by controlling the expression, activation, and phosphorylation of multiple targets, including CK2, caspase-9/3, DR-4, STAT-3/5, eIF-2α, Mcl-1, and HIF-1α. This work suggests that CX-4945 could be a promising drug for treating human CCA.

## 4. Materials and Methods

### 4.1. Chemicals and Antibodies

CX-4945 was purchased from Cayman Chemical (Ann Arbor, MI, USA). RPMI 1640 (LM-011-60), fetal bovine serum (FBS) (S001-01), and cocktail of penicillin/streptomycin cocktail (LS202-02) were obtained from Welgene, Inc. (Daegu, Republic of Korea). Control siRNA (cat. no. sc-37007), CK2α siRNA (cat. no. sc-29918), HIF-1α siRNA (cat. no. sc-35561), STAT-3 siRNA (cat. no. sc-29493), and STAT-5 siRNA (cat. no. sc-29495) were purchased from Santa Cruz Biotechnology (Delaware, CA, USA). A detailed list of antibodies used in this study is included in Supplementary Table S1. Western Bright^TM^ Enhanced chemiluminescence (ECL, cat. no. K-12045-D20) was purchased from Advansta Corporation (San Jose, CA, USA). All the plasticwares used for cell culture were obtained from SPL Life Sciences (Gyeonggi-do, Korea).

### 4.2. Cell Culture

Human cholangiocarcinoma cells HuCCT-1(JCRB0425, OUCI (JCRB), Shingo, Higashi-Matsuyama City, Japan), SNU1196 (KCLB01196, KCLB, Seoul, Korea) and human dermal fibroblasts (HDFs) (CL-173™, ATCC, Manassas, VA, USA) were grown in RPMI-1640 and DMEM with 10% heat-inactivated FBS (HI-FBS) and 1% penicillin/streptomycin at 37 °C in a humidified air (95% air O_2_ and 5% CO_2_), respectively.

### 4.3. Cell Count Analysis

HuCCT-1 cells were seeded in a density of 0.3 × 10^5^ cells/500 μL/well in 24-well plate overnight. Cells were treated with vehicle control (DMSO; 0.1%), CX-4945 or AG490 at the indicated concentrations and times. At each time point, the number of surviving cells, based on the principle that live cells have intact cell membrane which cannot be stained with trypan blue dye (0.4%, cat. no. 15250-061, Gibco, Grand Island, NY, USA), were counted using a phase-contrast microscope. Approximately 100 cells were counted for each evaluation. The cell survival is expressed as a percentage of control. Phase-contrast images of the conditioned cells were also taken with a compound microscope (Nikon Eclipse TS200, Nikon Corp., Tokyo, Japan).

### 4.4. Colony Formation Assay

HuCCT-1 cells were seeded at a density of 200 cells/0.5 mL/well in 24-well plate the day before treatment. Cells were treated with vehicle control (DMSO) or CX-4945 (20 μM) for two weeks. Colonies were fixed with 100% methanol and stained with 0.5% crystal violet [43].

### 4.5. Measurement of Genomic DNA Fragmentation

Measurement of genomic DNA fragmentation was conducted as previously described [44]. Briefly, HuCCT-1 cells were seeded at a density of 2.1 × 10^6^ cells/7 mL/100 mm plate the day before treatment. Cells were treated with vehicle control (DMSO) or CX-4945 at 5, 10, and 20 µM for 24 h. The conditioned cells were harvested, washed, and lysed in a buffer [50 mM Tris (pH 8.0), 0.5% sarkosyl, 0.5 mg/mL proteinase K, and 1 mM EDTA] at 55 °C for 3 h. Subsequently, RNase A at 0.5 μg/mL was added into the cell lysate and the cell lysate was further incubated at 55 °C for 18 h. The cell lysate was then centrifuged at 10,000× *g* at 4 °C for 20 min (min). Genomic DNA was obtained from the cell lysate by extracting with equal volume of neutral phenol-chloroform-isoamyl alcohol mixture (25:24:1) and analyzed via electrophoresis at 100 V on a 1.8% agarose gel for 20 min. The DNA was visualized and photographed under UV illumination after staining with ethidium bromide (0.1%, Sigma-Aldrich; Merck; St. Louis, MO, USA) using a gel documentation system (Gel Doc-XR, Bio-Rad Laboratories, Inc., Hercules, CA, USA).

### 4.6. Preparation of Protein Samples

HuCCT-1 cells were plated at a density of 6 × 10^5^ cells/2 mL/well in 6-well plate the day prior to treatment. After treatment, whole-cell lysates (WCL) were extracted from the conditioned cells using a modified RIPA buffer [50 mM Tris-Cl (pH 7.4), 150 mM NaCl, 0.1% sodium dodecyl sulfate, 0.25% sodium deoxycholate, 1% Triton X-100, 1% Nonidet P-40, 1 mM EDTA, 1 mM EGTA, proteinase inhibitor cocktail (1×)]. WCL was collected and centrifuged at 12,074× *g* for 20 min at 4 °C. The resultant supernatant containing proteins was saved and its protein concentration was determined using a bicinchoninic acid protein assay kit (Thermo Fisher Scientific, Inc., Rockford, IL, USA).

### 4.7. Immunoblot Analysis

An equal amount of proteins (40 μg) was separated via SDS-PAGE and transferred onto polyvinylidene fluoride (PVDF) membrane (Millipore; Billerica, MA, USA) by electroplating. The membranes were washed with Tris-buffered saline (TBS) (10 mM Tris, 150 mM NaCl, pH 7.5) supplemented with 0.05% (vol/vol) Tween 20 (TBS-T) followed by blocking with TBS-T containing 5% (wt/vol) non-fat dried milk. The membranes were probed overnight with primary antibodies at 4 °C, followed by incubation with secondary antibodies conjugated to horseradish peroxidase at room temperature for 2 h. The membranes were washed, and immune reactivities were detected using enhanced chemiluminescence reagents (cat. no. K 12045-D50; Advansta, Inc.). Equal protein loading was assessed by expression levels of control β-actin protein.

### 4.8. Small Interfering RNA (siRNA) Transfection

HuCCT-1 cells were seeded at a density of 1 × 10^5^ cells/2 mL/well in 6-well plate the day before transfection. Cells were transfected with 200 picomoles (pM) of each siRNA of control, CK2α, STAT-3, STAT-5 or HIF-1α using Lipofectamine^®^ RNAiMAX Transfection Reagent (Invitrogen, Waltham, MA, USA) in MEM media with not FBS for 6 h. The transfected cells were then grown in fresh RPMI media adjusted to be 10% HI-FBS as a final concentration for additional 36 h. After 48 h post-transfection, the numbers of surviving cells, which cannot be stained with trypan blue dye, were counted under microscope. For the measurement of each siRNA transfection efficiency, WCL from the transfected cells with respective siRNA was prepared and analysed by Western blotting.

### 4.9. Reverse Transcription-Polymerase Chain Reaction (RT-PCR)

After treatments, total cellular RNA was isolated using TRIzol reagent (Life Technologies, Carlsbad, CA, USA) according to the manufacturer’s protocol. Complementary DNA was then prepared using M-MLV reverse transcriptase (Gibco-BRL) according to the manufacturer’s protocol. Three micrograms of total RNA were transcribed and the cDNA prepared above was amplified by PCR with the primers listed in Table S2. Levels of actin mRNA expression were used as an internal control.

### 4.10. Small Interfering RNA (siRNA) Transfection

HuCCT-1 cells were seeded at a density of 1 × 10^5^ cells/2 mL/well in 6-well plate the day before transfection. Cells were transfected with 200 picomoles (pM) of each siRNA of control, CK2α, STAT-3, STAT-5 or HIF-1α using Lipofectamine^®^ RNAiMAX Transfection Reagent (Invitrogen, Waltham, MA, USA) in MEM media with not FBS for 6 h. The transfected cells were then grown in fresh RPMI media adjusted to be 10% HI-FBS as a final concentration for additional 36 h. After 48 h post-transfection, the numbers of surviving cells, which cannot be stained with trypan blue dye, were counted under microscope. For the measurement of each siRNA transfection efficiency, WCL from the transfected cells with respective siRNA was prepared and analysed by Western blotting.

### 4.11. Statistical Analyses

Cell count analysis was performed in triplicate. Data are expressed as mean ± standard error (SE) for at least three independent experiments. One-way ANOVA followed by Dunnett’s post hoc test was performed using SPSS 11.5 software (SPSS, Inc., Chicago, IL, USA). *p* < 0.05 was considered statistically significant.

## Data Availability

Data is contained within the article.

## Supplementary Materials

The following supporting information can be downloaded at: https://www.mdpi.com/article/10.3390/ijms23116353/s1.
