# Supplementary material for: Anti-Growth, Anti-Angiogenic, and Pro-Apoptotic Effects by CX-4945, an Inhibitor of Casein Kinase 2, on HuCCT-1 Human Cholangiocarcinoma Cells via Control of Caspase-9/3, DR-4, STAT-3/STAT-5, Mcl-1, eIF-2α, and HIF-1α
Source: Int J Mol Sci. 2022 Jun 6;23(11):6353. doi: 10.3390/ijms23116353 (PMC9181600; doi:10.3390/ijms23116353)
Supplement: Supplementary file 1 [file ijms-23-06353-s001.zip › ijms-1669020 Supplementary Figures S1 (A, B) and S2.pptx]

## Slide 1
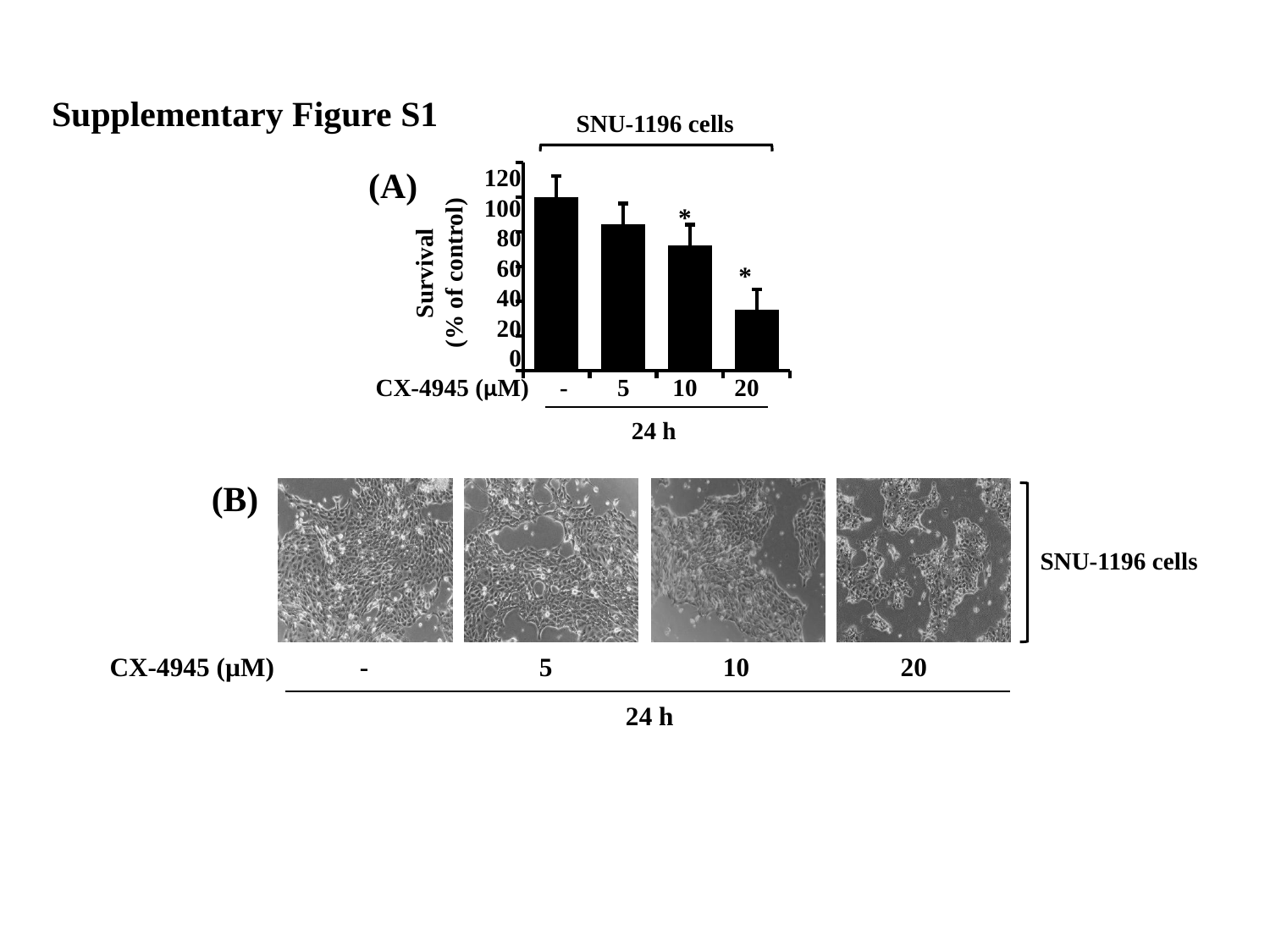

Supplementary Figure S1
SNU-1196 cells
(A)
### Chart
| Category | |
|---|---|120
100
80
60
40
20
0
*
Survival
(% of control)
*
CX-4945 (μM) - 5 10 20
24 h
(B)
SNU-1196 cells
 CX-4945 (µM) - 5 10 20
24 h

## Slide 2
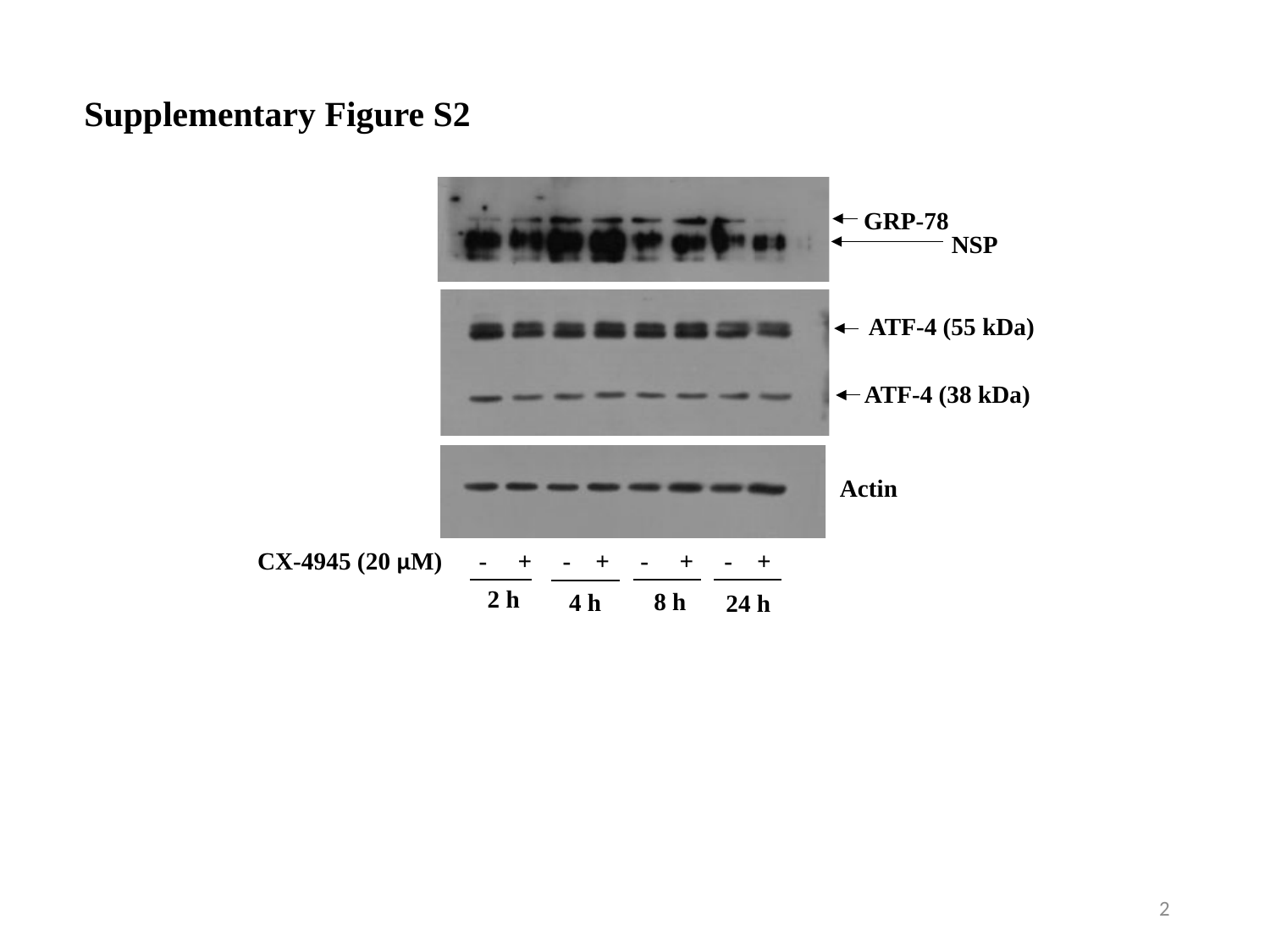

Supplementary Figure S2
GRP-78
NSP
ATF-4 (55 kDa)
ATF-4 (38 kDa)
Actin
CX-4945 (20 μM) - + - + - + - +
2 h
8 h
4 h
 24 h
2
