# Supplementary material for: Anti-Growth, Anti-Angiogenic, and Pro-Apoptotic Effects by CX-4945, an Inhibitor of Casein Kinase 2, on HuCCT-1 Human Cholangiocarcinoma Cells via Control of Caspase-9/3, DR-4, STAT-3/STAT-5, Mcl-1, eIF-2α, and HIF-1α
Source: Int J Mol Sci. 2022 Jun 6;23(11):6353. doi: 10.3390/ijms23116353 (PMC9181600; doi:10.3390/ijms23116353)
Supplement: Supplementary file 1 [file ijms-23-06353-s001.zip › ijms-1669020 Table (supple. doc.pdf]

Table S1: List of antibodies used for Western blot analysis.

Antibodies	Dilution used	Source	Catalog no.
Primary antibodies			
Procaspase-3	1:2,000	Enzo Life Sciences	ADI-AAP-113
Procaspase-9	1:2000	Enzo Life Sciences	ADI-AAP-139
PARP	1:2,000	Cell signalling	#9532
DR-4	1:2,000	Santa Cruz Biotechnology	sc-8411
p-CK2α	1:2,000	Sigma	111411572
CK2α	1:2,000	Cell signalling	#2656
p-CK2 substrates	1:2,000	Cell signalling	#8738
p-eIF-2α (S51)	1:2,000	Abcam	ab32157
eIF-2α	1:2,000	Cell signalling	#9722
p-STAT-3 (Y705)	1:2,000	Santa Cruz Biotechnology	sc-8059
STAT-3	1:2,000	Santa Cruz Biotechnology	sc-8019
p-STAT-5	1:2,000	Santa Cruz Biotechnology	sc-101806
STAT-5	1:2,000	Santa Cruz Biotechnology	sc-835
HIF-1α	1:2,000	BD Biosciences	610958
HIF-1β	1:2,000	Santa Cruz Biotechnology	sc-17811
Mcl-1	1:2,000	Santa Cruz Biotechnology	sc-819
β-Actin	1:10,000	Sigma	A5441
Secondary antibodies			
Goat anti-rabbit IgG-HRP	1:5000	Jackson ImmunoResearch Laboratories	111-035-045
Goat anti-mouse-IgG-HRP	1:5000	Jackson ImmunoResearch Laboratories	115-035-062

Table S2: Sequences of primers used for RT-PCR.

Gene	Forward	Reverse
STAT-3	CAAAACCCTCAAGAGCCAAG	TCACTCACAATGCTTCTCCG
Mcl-1	ATCTCTCGGTACCTTCGGGAG	ACCAGCTCCTACTCCAGCAAC
HIF-1α	CTCAAAGTCGGACAGCCTCA	CCCTGCAGTAGGTTTCTGCT
HIF-1β	GTGCGCACACATGCTTCTGT	CTTTATGGCCAAGTCTCGGGT
Actin	TCAAGATCATTGCTCCTCCTG	CTGCTTGCTGATCCACATCTG
